# Modeling gene interactions in polygenic prediction via geometric deep learning

**DOI:** 10.1101/gr.279694.124

**Published:** 2025-01

**Authors:** Han Li, Jianyang Zeng, Michael P. Snyder, Sai Zhang

**Affiliations:** 1 School of Mathematical Sciences and LPMC, Nankai University, Tianjin, 300071, China;; 2 Institute for Interdisciplinary Information Sciences, Tsinghua University, Beijing, 100084, China;; 3 School of Engineering, Research Center for Industries of the Future, Westlake University, Hangzhou, 310030, Zhejiang, China;; 4 Department of Genetics, Center for Genomics and Personalized Medicine, Stanford University School of Medicine, Stanford, California 94304, USA;; 5 Department of Epidemiology, University of Florida, Gainesville, Florida 32603, USA;; 6 Departments of Biostatistics & Biomedical Engineering, UF Genetics Institute, University of Florida, Gainesville, Florida 32603, USA

## Abstract

Polygenic risk score (PRS) is a widely used approach for predicting individuals’ genetic risk of complex diseases, playing a pivotal role in advancing precision medicine. Traditional PRS methods, predominantly following a linear structure, often fall short in capturing the intricate relationships between genotype and phenotype. In this study, we present PRS-Net, an interpretable geometric deep learning–based framework that effectively models the nonlinearity of biological systems for enhanced disease prediction and biological discovery. PRS-Net begins by deconvoluting the genome-wide PRS at the single-gene resolution and then explicitly encapsulates gene–gene interactions leveraging a graph neural network (GNN) for genetic risk prediction, enabling a systematic characterization of molecular interplay underpinning diseases. An attentive readout module is introduced to facilitate model interpretation. Extensive tests across multiple complex traits and diseases demonstrate the superior prediction performance of PRS-Net compared with a wide range of conventional PRS methods. The interpretability of PRS-Net further enhances the identification of disease-relevant genes and gene programs. PRS-Net provides a potent tool for concurrent genetic risk prediction and biological discovery for complex diseases.

Complex human diseases exhibit substantial polygenicity in their genetic architectures, characterized by a multitude of common genetic variants with moderate or minor effects accumulatively influencing the disease risk (Clarke et al. 2009; CKDGen Consortium et al. 2011; Orrù et al. 2013; Gaiteri et al. 2016). Polygenic risk scores (PRSs) (Torkamani et al. 2018; Choi et al. 2020; Lewis and Vassos 2020), also known as polygenic scores (PGSs), have been developed to quantitatively estimate the genetic susceptibility of individuals to specific traits or diseases based on common variants (i.e., variants with minor allele frequency [MAF] < 0.05 in the population). This methodology empowers several aspects of precision medicine, including disease prevention, early intervention, and personalized treatment (Gibson 2019; Konuma and Okada 2021; Polygenic Risk Score Task Force of the International Common Disease Alliance 2021).

PRS is calculated based on summary statistics derived from the genome-wide association study (GWAS) (Euesden et al. 2015; Vilhjálmsson et al. 2015; Mak et al. 2017; Choi and O'Reilly 2019; Lloyd-Jones et al. 2019; Privé et al. 2021, 2022), a widely used statistical method for identifying disease-associated genetic variants (Wang et al. 2005; Korte and Farlow 2013; Uffelmann et al. 2021). Although GWAS enables genome-wide identification of risk variants, such as single-nucleotide polymorphisms (SNPs) and small insertions and deletions (indels), which exhibit significant differences in allele frequency between disease cases and healthy controls, these GWAS hits tend to have modest or even minor effects on the phenotype, resulting in limited prediction capability for individuals. In an effort to enhance predictive modeling, various statistical methods have been proposed to aggregate variants with a wide range of significance (e.g., GWAS *P* < 5 × 10^−8^), such as the clumping and thresholding (C + T) method (Purcell et al. 2007; Vilhjálmsson et al. 2015), PRSice-2 (Euesden et al. 2015), LDpred2 (Privé et al. 2021), and lassosum2 (Privé et al. 2022). However, these approaches mainly follow an additive structure and oversimplify the intricate relationship between genotype and phenotype.

It has been broadly recognized that human phenotype is determined not solely by single genes but rather complex interactions among multiple genes or proteins, exhibiting additive or nonadditive genotype–phenotype (G2P) relationships (Cordell 2009; Zuk et al. 2012; Taylor and Ehrenreich 2015). Importantly, there is increasing evidence highlighting the significance of nonadditive G2P (Lenz et al. 2015; Varona et al. 2018; Guindo-Martínez et al. 2021). Epistasis is a prominent example that occurs when the impact of a gene variant depends on the presence or absence of variants in other genes (Cheverud and Routman 1995; Moore and Williams 2009; Lehner 2011). Several efforts have been made to consider such nonlinear genetic interactions in PRS calculation. These include tree-based methods such as random forests (Ho 1995; Breiman 2001), gradient boosting (Friedman 2001, 2002), and AdaBoost (Freund and Schapire 1997; Hastie et al. 2009), as well as deep learning–based models such as multiple-layer perceptrons (MLP) (Alzoubi et al. 2023) and convolutional neural networks (Sigurdsson et al. 2023). However, these methods only take a limited number of variants as their input and also lack the integration of prior knowledge regarding biological complexity. Indeed, existing nonlinear PRSs have demonstrated either comparable or, in many cases, inferior performance in predicting phenotypes compared with linear models (Bellot et al. 2018; Xu et al. 2022).

In this study, we propose PRS-Net, a geometric deep learning–based framework tailored to effectively model nonlinear relationships among genetic factors, thus delivering more accurate and robust genetic risk prediction. Based on the GWAS summary statistics, PRS-Net first maps the genome-wide PRS onto a gene–gene interaction (GGI) network through the derivation of gene-level PRSs using the C + T method. Subsequently, a graph neural network (GNN) is employed to iteratively update the PRS embeddings of genes via message passing. An attentive readout module is introduced to enhance model interpretability. PRS-Net also integrates a mixture-of-expert module (Masoudnia and Ebrahimpour 2014) to improve prediction generalization across multiancestry data sets. Based on the UK Biobank (UKBB) database (Sudlow et al. 2015), extensive evaluations were performed for eight diseases—including Alzheimer's disease (AD), atrial fibrillation (AF), rheumatoid arthritis (RA), multiple sclerosis (MS), ulcerative colitis (UC), asthma, myocardial infarction (MI), and coronary artery disease (CAD)—and two traits, including height and body mass index (BMI). PRS-Net exhibited superior performance in predicting both diseases and traits compared with linear baseline methods, including PLINK C + T, PRSice-2, LDpred2, and lassosum2, as well as nonlinear models, including MLP and XGBoost (Chen and Guestrin 2016). Notably, we demonstrated the portability of PRS-Net across diverse ancestries. Through model interpretation, we further showcased the enhanced capacity of PRS-Net in pinpointing disease-relevant genes and gene programs. PRS-Net provides a potent framework for precise genetic risk prediction and systematic discovery of genetic and molecular underpinnings of complex traits and diseases.

## Results

### Overview of PRS-Net

**Figure 1.**
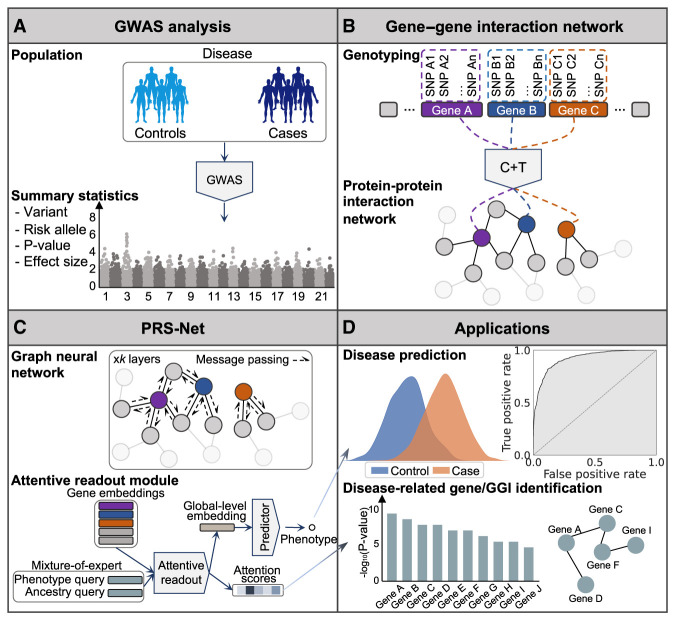
An illustrative diagram of PRS-Net. (*A*) The proposed framework is built upon GWAS summary statistics, including variants, risk alleles, *P*-values, and effect sizes. (*B*) A gene–gene interaction (GGI) network is constructed based on the protein–protein interactions (PPIs) in this study. Gene-level PRSs (various *P*-value thresholds applied) are calculated using the C + T method, serving as the node features within the network. (*C*) A graph neural network (GNN) is employed to update node features via message passing, and subsequently, an attentive readout module is introduced to facilitate model interpretation. (*D*) PRS-Net can be applied for both disease prediction and gene discovery. (GWAS) genome-wide association stuty, (C + T) clumping and thresholding.

PRS-Net is a deep learning framework designed to enhance PRS prediction and gene discovery by modeling gene interactions using a GNN model (Methods). First, PRS-Net calculates gene-level PRSs based on GWAS summary statistics using a C + T method implemented by PLINK (Fig. 1A,B; Purcell et al. 2007; Vilhjálmsson et al. 2015). This method focuses on genetic variants within and near the gene body, encompassing both coding and noncoding genomic regions. By applying multiple *P*-value thresholds, several PRS values are generated for each gene, which then serves as features for corresponding genes. To characterize gene interactions in the biological system, PRS-Net incorporates a protein–protein interaction (PPI) network constructed from the STRING database (Fig. 1B; Szklarczyk et al. 2023). This network integration enables PRS-Net to capture high-order relationships between genotype and phenotype.

Next, gene-level PRSs and the PPI network are fed into a graph isomorphism network (GIN) (Fig. 1C; Xu et al. 2018), by which the gene features are updated iteratively by aggregating information from neighboring genes. Additionally, we adopt an attentive readout module to compute the global representation for each individual based on the attention mechanism, wherein the attention scores indicate gene importance to the phenotype of interest. This global representation is then used for making the final prediction. To address the challenge of cross-ancestry prediction, PRS-Net also incorporates a mixture-of-expert module (Masoudnia and Ebrahimpour 2014) with ancestry-specific attention modules.

By integrating genetic data with biological network priors, PRS-Net offers as a powerful tool to the community with diverse downstream applications, ranging from disease prediction to biological discovery (Fig. 1D). In this study, the UKBB was used as the primary database for model training and testing (Methods). PRS-Net utilizes two cohorts: the base cohort analyzed in GWAS to estimate per-variant effect sizes and *P*-values (Fig. 1A), and the target cohort used to train and test PRS-Net. To prevent information leakage, we selected GWAS independent with UKBB for each disease and trait.

### PRS-Net improves disease risk prediction

**Figure 2.**
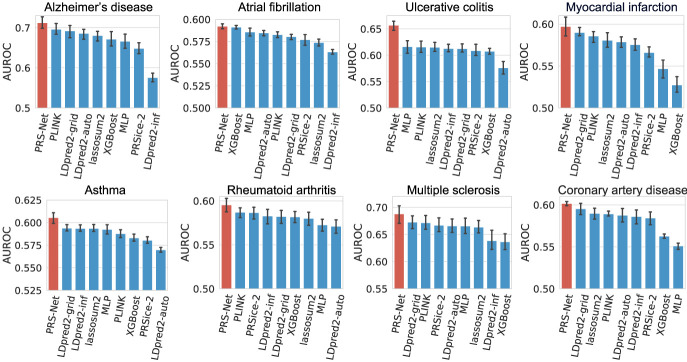
Prediction performance evaluation based on the area under the receiver operating characteristic curve (AUROC) for different diseases (41,175 test samples in total). The bar plot and error bar denote the mean and standard error, respectively. The training, validation, and testing procedure was conducted for six repeats with different random seeds for each model and each disease.

We first benchmarked PRS-Net against multiple existing PRS methods in disease risk prediction (Methods) (Supplemental Table S1). Here, only UKBB Western European (EUR) samples were included in the analysis. Both metrics including the area under the receiver operating characteristic (AUROC) and the area under the precision-recall curve (AUPRC) were adopted for performance evaluation. Our results revealed that PRS-Net consistently outperformed all baseline methods, including nonlinear methods for all diseases, yielding relative improvements ranging from 0.7% to 6.9% in AUROC (Fig. 2) and 1.3% to 18.3% in AUPRC (Supplemental Fig. S1). Importantly, tests on two autoimmune diseases achieved the largest improvements, including UC (6.9% in AUROC and 18.3% in AUPRC) and MS (2.2% in AUROC and 8.4% in AUPRC), reinforcing the observed nonadditivity of genomic factors underlying these diseases (Lipsitch et al. 2003; Tsai and Santamaria 2013; Goyette et al. 2015; Lenz et al. 2015).We also compared PRS-Net with the best linear unbiased prediction (BLUP) using BOLT-LMM-inf (Loh et al. 2015) to generate the BLUP estimates, confirming the superiority of PRS-Net (Supplemental Fig. S2).

To understand the suboptimal performance yielded by nonlinear baseline models, we developed two alternative versions of MLP and XGBoost, referred to as MLP_less_snp and XGBoost_less_snp, which only utilized GWAS variants included in PRS-Net (Supplemental Methods). We observed MLP_less_snp and XGBoost_less_snp displayed a performance comparable or even superior to that of the original MLP and XGBoost models (Supplemental Fig. S3). This suggests that existing nonlinear approaches may fail to effectively learn disease-variant associations.

**Figure 3.**
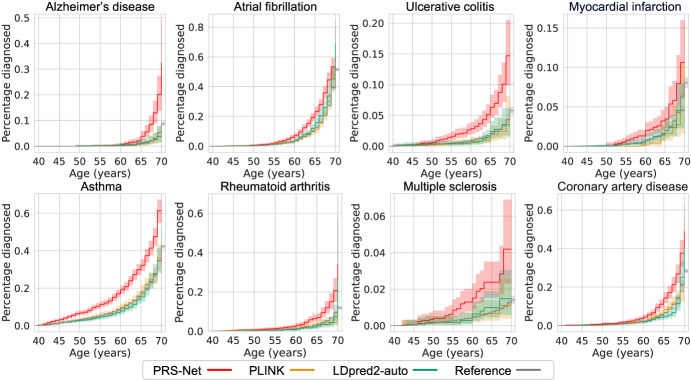
The cumulative incidence plots of high-risk individuals (with the highest 5% PRSs) identified by PRS-Net and baseline methods. Each plot illustrates the estimated percentage of individuals diagnosed with a specific disease at different ages. We provide cumulative incidence plots for the original data sets as references. The shaded area denotes the standard error estimated based on six repeats.

We next utilized the Aalen–Johansen estimator (Aalen and Johansen 1978) to calculate the disease occurrence over the lifetime for individuals stratified into high-risk or low-risk groups, as determined by different PRS methods. High-risk individuals were defined as those with the highest 5% of PRSs, whereas low-risk individuals were identified as those with the lowest 5% of PRSs. High-risk individuals defined by PRS-Net exhibited an elevated risk of disease throughout their lifetime compared with those predicted by baseline methods, especially for UC, asthma, RA, and MS (Fig. 3). Meanwhile, low-risk individuals categorized by PRS-Net tended to maintain a lower risk of disease (Supplemental Fig. S4). These results underscore the potential of PRS-Net as an effective tool for disease risk stratification.

### PRS-Net enhances quantitative trait prediction

**Figure 4.**
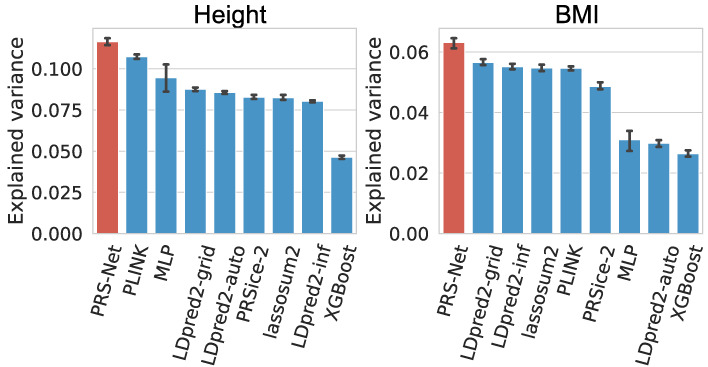
Prediction performance evaluation for quantitative traits (41,028 and 40,411 test samples for height and body mass index [BMI], respectively). Performance was measured in explained variance. The bar plot and error bar denote the mean and standard error, respectively. The training, validation, and testing procedure was conducted for six repeats with different random seeds for each model and each trait.

We further applied PRS-Net on two quantitative traits, including height and BMI. Similarly, we focused on UKBB EUR samples in the analysis. Explained variance and *R*^2^ were used for performance evaluation (Methods). Again, PRS-Net exhibited superior prediction performance compared with all baseline methods for both traits (Fig. 4; Supplemental Fig. S5), with relative improvements of 8.50% and 11.64% for height and BMI in terms of explained variance, respectively. This highlights the generalizability of PRS-Net in predicting diverse phenotypes.

### PRS-Net boosts cross-ancestry prediction

**Figure 5.**
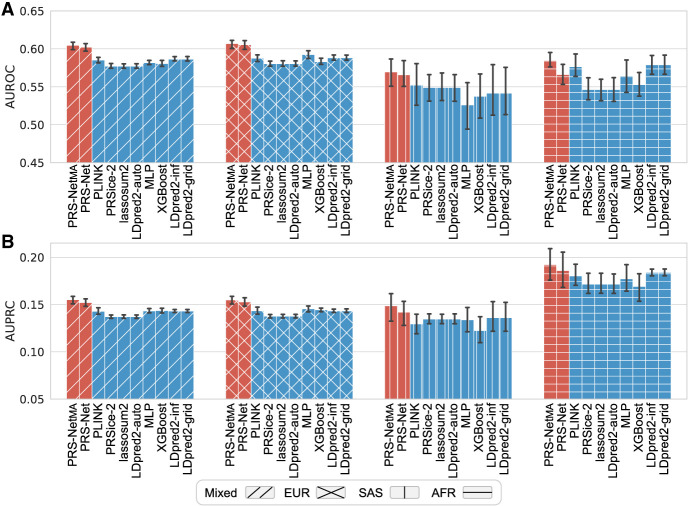
Prediction performance evaluation for asthma across multiple populations, including Western European (EUR), South Asian (SAS), and African (AFR) ancestries, measured by the area under receiver operating characteristic curve (AUROC; *A*) and the area under precision-recall curve (AUPRC; *B*), respectively. The results on the mixed ancestry test set were also reported. The bar plot and error bar denote the mean and standard error, respectively. The training, validation, and testing procedure was conducted for six repeats.

Next, we assessed the performance of PRS-Net and our multiple-ancestry model, PRS-Net_MA_, on the data set comprising individuals from diverse ancestral backgrounds. Specifically, we curated a mixed-ancestry UKBB data set encompassing EUR, South Asian (SAS), and African (AFR) for asthma, which contained a reasonable number of asthma cases (*N* > 1000) for each ancestry group (Supplemental Table S2). PRS-Net, trained solely on EUR samples, still outperformed baseline methods on the mixed and SAS test sets, suggesting that PRS-Net captured disease biology independently of ancestral backgrounds (Fig. 5). PRS-Net_MA_ further improved cross-ancestry prediction and yielded a superior or comparable performance compared with PRS-Net on these ancestry-specific test sets (Fig. 5). These results demonstrate the portability of PRS-Net and PRS-Net_MA_ across diverse ancestral groups in genetic risk prediction.

### PRS-Net identifies disease-relevant genes and GGIs

Following the demonstration on the superiority of PRS-Net in disease prediction, we sought to examine its capability in gene discovery. Specifically, we applied the Mann–Whitney *U* test (Mann and Whitney 1947) to each gene assessing whether its attention scores for disease cases were significantly higher than those of healthy controls. For AD, this analysis yielded a gene set comprising 142 significant genes (adjusted *P* < 0.05, Bonferroni correction) (Supplemental Table S3). Gene set enrichment analysis (GSEA) (Subramanian et al. 2005) presented the enrichment of lipoprotein particles for PRS-Net-prioritized genes (Supplemental Fig. S6). This observation is in line with prior studies implicating lipoprotein particles as a risk factor of AD (Safieh et al. 2019; Zhou et al. 2020; Jin et al. 2021), with therapeutic potentials (Safieh et al. 2019; Jin et al. 2021).

**Figure 6.**
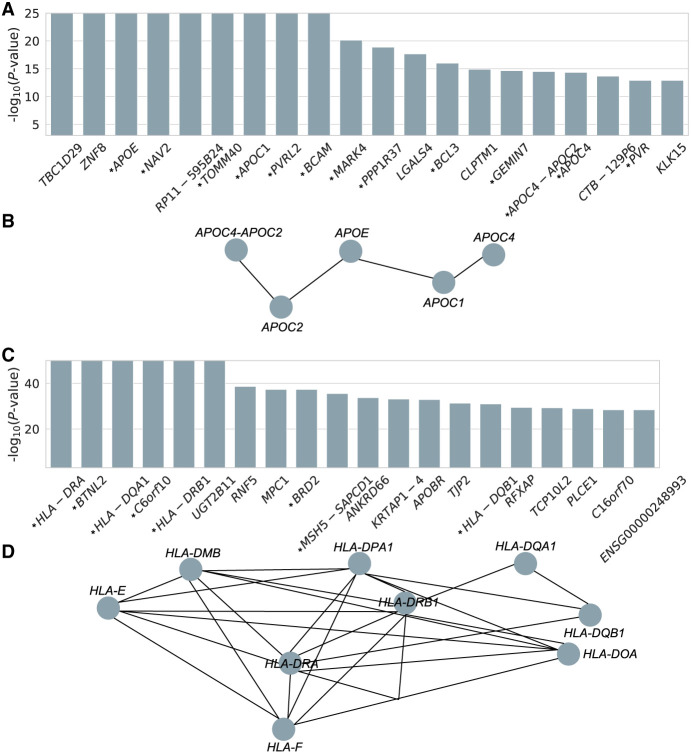
PRS-Net identifies disease-relevant genes and gene modules for Alzheimer's disease and multiple sclerosis. (*A*) Top 20 genes ranked by *P*-values for Alzheimer's disease. *P*-value by the Mann–Whitney *U* test. An asterisk preceding the gene name signifies this gene has been reported to be associated with Alzheimer's disease in previous studies. (*B*) An example of PPIs among PRS-Net-identified genes for Alzheimer's disease. (*C*) Top 20 genes ranked by *P*-values for multiple sclerosis. (*D*) An example of PPIs among PRS-Net-identified genes for multiple sclerosis.

From the results, 15 out of the top 20 genes (ranked by *P*-values) (Fig. 6A) have been identified as AD risk factors in previous studies. One notable example is APOE, which is the most prevalent high-density lipoprotein in the central nervous system (CNS) and has been consistently linked to AD in the literature (Tsai et al. 1994; Strittmatter and Roses 1995; Meyer et al. 1998; Green et al. 2009; Genin et al. 2011; Yamazaki et al. 2019). Other well-recognized AD genes interacting with APOE, including *APOC1*, *APOC2*, and *APOC4*, were also prioritized by PRS-Net (Fig. 6B).

For MS, our analysis identified 154 significant risk genes (adjusted *P* < 0.05, Bonferroni correction) (Supplemental Table S4). GSEA of PRS-Net-identified genes highlighted numerous immune-related pathways and the major histocompatibility complex (MHC) protein complex (Supplemental Fig. S7), agreeing well with the literature (Traugott 1987; Lee et al. 1990; Multiple Sclerosis Genetics Group et al. 1998; Dyment et al. 2005; Lincoln et al. 2005). A considerable number of *HLA* genes were prioritized by PRS-Net (Fig. 6C,D), reinforcing previous studies indicating that HLA interactions modulate the genetic risk of MS (The International Multiple Sclerosis Genetics Consortium 2015). Additionally, nonadditive interactions between HLAs have been widely reported to significantly affect the risk of autoimmune diseases (Lipsitch et al. 2003; Tsai and Santamaria 2013; Goyette et al. 2015; Lenz et al. 2015).

To examine the contribution of GGIs in gene discovery, we trained a PRS-Net variation without the GGI network, denoted as PRS-Net-noPPI, and then applied it to identify disease genes using the same strategy. PRS-Net-noPPI identified a much smaller number of genes (*N* = 3 for AD and *N* = 1 for MS) (Supplemental Fig. S8) compared with PRS-Net. This supports the importance of GGIs in increasing the power of discovering disease genes.

### Ablation studies

**Figure 7.**
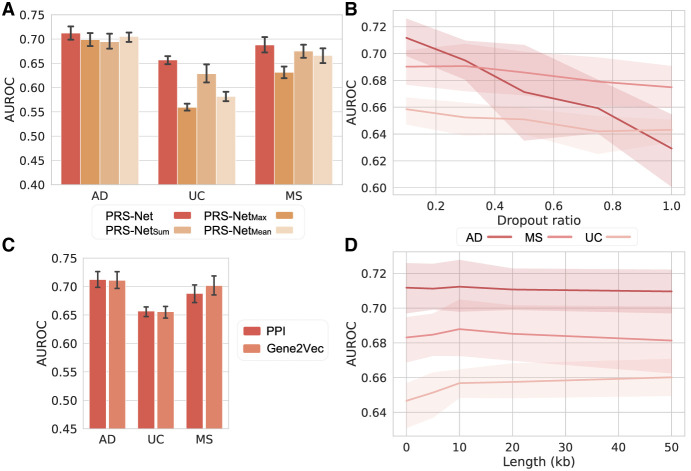
The ablation results for PRS-Net. (*A*) The performance comparison between PRS-Net and its multiple variations. The bar plot and error bar denote the mean and standard error, respectively. (*B*) The performance of PRS-Net with PPI dropout. The line plot and shaded area denote the mean and standard error, respectively. (*C*) Comparison results of PRS-Net with different GGI networks. (*D*) The prediction performance of PRS-Net with different extension lengths. (AD) Alzheimer’s disease, (MS) multiple sclerosis, (UC) ulcerative colitis, (AUROC) the area under the receiver operating characteristic curve, (PPI) protein–protein interaction.

To examine the contribution of different components in PRS-Net, we conducted extensive ablation studies. We first introduced multiple variations of PRS-Net, including PRS-Net_Sum_, PRS-Net_Mean_, and PRS-Net_Max_, replacing the attentive readout module with summation, averaging, and maximization operations, respectively. PRS-Net outperformed PRS-Net_Sum_, PRS-Net_Mean_, and PRS-Net_Max_ (Fig. 7A; Supplemental Fig. S9A), with average relative improvements of 3.0%, 9.4%, and 5.7% in AUROC, respectively. This highlights the critical role of the attention module in boosting prediction performance.

Next, we assessed the impact of the GGI network on prediction. We tested it by randomly dropped PPIs with different ratios. As shown in Figure 7B and Supplemental Figure S9B, we observed a continuous decrease in prediction performance as the dropout ratio increased across different diseases, validating the contribution of GGIs in disease prediction. We also examined the impact of different PPI filtering thresholds on the prediction performance. PRS-Net yielded a comparable performance when we increased the threshold to 0.9 (Supplemental Fig. S10A); however, lowering the threshold below 0.8 resulted in a significant drop in performance. We further randomized the PPI network and, as expected, noticed a substantial performance decrease (Supplemental Fig. S10B). These results together highlight the contribution of a well-defined PPI network on the robustness and effectiveness of PRS-Net.

Subsequently, we evaluated the performance of PRS-Net with different GGI networks. Specifically, we employed gene representations generated by Gene2vec (Du et al. 2019). This machine learning approach leverages gene coexpression information extracted from 984 NCBI Gene Expression Omnibus (GEO; https://www.ncbi.nlm.nih.gov/geo/) (Barrett et al. 2012) data sets to infer gene embeddings capturing the functional similarity between genes. We constructed a *k*-nearest neighbors graph based on the cosine similarity between gene embeddings as an alternative GGI network. We set *k* = 15 to match the number of interactions in our original PPI setting. We found that PRS-Net equipped with either GGI networks exhibited comparable prediction performance (Fig. 7C; Supplemental Fig. S9C), highlighting the robustness of PRS-Net against different GGI networks.

In our model design, we extended gene bodies to incorporate genetic variants upstream of and downstream from individual genes. In addition to coding regions, these extended gene bodies (>20 kb) also covered noncoding regulatory regions, such as promoters and enhancers, which control the expression levels of target genes. We further explored the impact of different extension lengths (i.e., 0 kb, 5 kb, 10 kb, 20 kb, and 50 kb) on prediction. In general, PRS-Net displayed stable performance across different extension lengths (Fig. 7D; Supplemental Fig. S9D). It is noteworthy that the performance on MS was significantly reduced when no extension was employed (Fig. 7D), implicating the benefit of incorporating regulatory variants in genetic risk prediction for certain diseases.

We further investigated how the gene extension length, in conjunction with *R*^2^ and clumping window size, impacts the model performance. In particular, we evaluated different combinations of *R*^2^ = 0.1 or 0.5, *L* = 0 kb or 10 kb, and window size = 25 kb or 250 kb, resulting in eight combinations in total. As shown in Supplemental Figure S11, the model performance was generally improved with a larger window size or gene extension length, whereas different values of *R*^2^ appeared to have a marginal effect on performance.

To assess the impact of nonlinearity introduced by the MLP, we conducted a comparison between PRS-Net with a linear predictor (PRS-Net-Linear) and with an MLP predictor (PRS-Net-MLP). As shown in Supplemental Figure S12, both models achieved comparable results across different diseases, implicating that although MLP introduced additional nonlinearity, it was not necessary for boosting the performance in general. This further highlights the importance of the GNN module in enhancing the overall genetic prediction.

## Discussion

In this study, we developed PRS-Net, a geometric deep learning–based framework that achieves in tandem disease prediction and biological discovery. By explicitly modeling GGIs using a GNN, PRS-Net enables the characterization of nonlinear relationships between genotype and phenotype. The integration of an attention module further improves model interpretability. Extensive tests on eight diseases and two quantitative traits demonstrate the superiority of PRS-Net over multiple baseline PRS methods in disease prediction and gene discovery.

A standard clumping and thresholding (C + T) PRS integrates not only genome-wide significant ($P<\;5\times 10^{-8}$) variants but also marginal (P<0.05) or even nonsignificant (P>0.05) ones to enhance the prediction performance (Choi et al. 2020). The *P*-value threshold, along with LD *R*^2^ threshold, determines the set of variants we want to include in a PRS model, as well as the best threshold varies for different diseases and traits. The design principle of PRS-Net is to decompose the genome-wide PRS into single-gene resolution to measure the contribution of each gene to the disease risk. This is achieved by calculating C + T PRS per gene and then integrating gene-level PRS features leveraging a GGI network. Because the best threshold is unknown in advance, here we chose to compute gene-level PRSs with multiple thresholds as the input node features and then integrated them using an embedding layer. This unbiased strategy enables us to assess the cumulative genetic contribution across various levels of statistical significance, capturing a broader spectrum of genetic information beyond top associations identified by GWAS. Although we have not experimented with other ways of initializing node features, we acknowledge the potential for further exploration in this aspect. We employed PLINK C + T, a most widely used method, to calculate gene-level PRSs in our implementation, but we recognize the possibilities of using alternative approaches, such as PRSice2, or even combinations of these approaches for gene-level PRS computation.

One of the key motivations of our work is to empower biological discovery via model interpretation, which is lacking in traditional GWAS and PRS. By mapping a wide range of variants to their nearby genes, which has been demonstrated effective in nominating disease genes (Fulco et al. 2019; Nasser et al. 2021), we calculated gene-level PRS and then conducted GNN operations at the gene level. This layer-wise strategy achieves biology-informed disease prediction (Elmarakeby et al. 2021; Zhang et al. 2024), enabling a systematic investigation of the underlying disease biology. Of note, the flexibility of our framework also allows for the incorporation of distal intergenic variants (i.e., extending gene bodies to their linked distal regulatory regions) as long as their target genes are well defined. However, we leave this to the future work given the current challenges in linking distal variants to genes (Schnitzler et al. 2024).

In the message passing phase of GNN, each node aggregates information from its neighboring nodes. In our context, each gene within the GGI network collects the genetic information from neighboring genes and refines its own features through this message passing process. This effect is desirable in our scenario because genes and proteins are not working in isolation but collaboratively to regulate various biological processes. The function of a gene may be compromised if the genes it interacts with experience a loss of function. This is exemplified by cases in which disease genes converge on particular gene modules or pathways (Berg and Geschwind 2012; Walker et al. 2023). Therefore, the propagation of PRS signals over GGIs facilitates the capture of inherent interdependencies among genes, contributing to a system understanding of disease etiology and enhanced risk prediction.

In our tests, we specifically opt for the GIN (Xu et al. 2018) owing to its proven theoretical and empirical expressiveness. From a theoretical perspective, the seminal GIN paper established a theoretical framework by connecting GNNs with the Weisfeiler–Lehman (WL) graph isomorphism test. The WL test iteratively updates node feature vectors by aggregating neighbor features, providing a powerful tool for distinguishing between different graph structures. This theoretical analysis demonstrates GIN's outstanding expressiveness and supports our choice of GIN for its ability in effectively capturing complex interactions among elements. From an empirical perspective, GIN has demonstrated its efficacy across various applications, including but not limited to molecular representation learning (Wang et al. 2022), compound–protein interaction prediction (Lin et al. 2022), and spatial cellular modeling (Wu et al. 2022). The consistent superior performance shown in these studies underscores GIN's capability in modeling diverse graph-structured biological data.

We reviewed several studies that reported the variance explained by polygenic models for height and BMI in the UKBB. Specifically, the variance explained is 1.5%, 1.6%, or 4.1% for BMI (Tyrrell et al. 2016; Sun et al. 2019; Vithayathil et al. 2021) and 5.5% or 12.3% for height (Tyrrell et al. 2016; Vithayathil et al. 2021), based on different studies. These data are consistent with our results. The performance of PRS models is considerably influenced by the discovery GWAS. In our tests, we utilized data from the GIANT consortium, a widely used standard database for height and BMI; however, it is important to note that the discrepancies between height GWAS from GIANT and from UKBB have been reported (Sohail et al. 2019), which could have contributed to the lower performance than expected. In addition, our initial analyses did not incorporate covariates such as age, sex, and principal components (PCs) into PRS modeling. When we included these covariates into the PRS-Net and baseline methods, we noticed a significant improvement across all methods (Supplemental Fig. S13).

To examine the capacity of PRS-Net in modeling nonlinearity between genotype and phenotype, we performed a simulation study. We first designed a simulation approach allowing us to simulate phenotypes that exhibit nonlinear relationships with genetic features. In detail, we utilized a PRS-Net model with fixed weights (randomly initialized) to generate quantitative traits for individuals, using gene-level features derived from the height GWAS summary statistics as input. Because PRS-Net inherently models protein–protein interactions, the simulated traits reflect a nonlinear function of the input genetic features. We then trained PRS-Net based on this simulation data set and compared it with linear regression mimicking traditional PRS. As shown in Supplemental Figure S14A, PRS-Net significantly outperformed the linear model in phenotype prediction, demonstrating its effectiveness in modeling nonlinear G2P. As a negative control, we also generated traits from genotypes using a linear model. Specifically, we used effect sizes (i.e., β’s) derived from the height GWAS to compute a weighted sum of individuals’ genotypes, yielding their simulated traits. PRS-Net presented comparable prediction performance with linear regression (Supplemental Fig. S14B). Altogether, our results demonstrate PRS-Net is well behaved in enhancing PRS prediction by capturing nonlinear gene interactions, showing no significant false-positive effect.

In summary, PRS-Net represents a novel biology-informed PRS framework, providing a potent tool for accurate genetic prediction and systematic biological discovery for complex traits and diseases.

## Methods

### Gene-level PRSs

We first compute gene-level PRSs using the PLINK C + T method (Fig. 1B; Purcell et al. 2007; Vilhjálmsson et al. 2015). In particular, for each gene we focus on the variants residing within the extended gene body spanning from *L* bp upstream of its transcription start site (TSS) to *L* bp downstream from the transcription end site. In our study, we set *L* = 10,000, thereby encompassing variants located within noncoding regulatory regions, such as promoters and enhancers. The linkage disequilibrium (LD) estimated based on the 1000 Genomes European samples (The 1000 Genomes Project Consortium 2015) is adopted to perform clumping and remove correlated variants, wherein we set *R*^2^ = 0.5 and window size = 250 kb. Given a specific *P*-value threshold, the gene-level PRSs utilizing all variants with GWAS *P*-values below this threshold are computed across all genes for each individual in the target cohort. In this study, we set multiple *P*-value thresholds including 1 × 10^−5^, 1 × 10^−4^, 1 × 10^−3^, 1 × 10^−2^, 5 × 10^−2^, 0.1, 0.2, 0.3, 0.4, 0.5, and one, yielding 11 PRSs for each gene.

### GGI network

We establish a GGI network that empowers PRS-Net to capture molecular interactions underlying the target phenotype (Fig. 1B). We construct our GGI network based on the high-confidence PPIs (with a confidence score *>* 0.8) derived from the STRING database (Szklarczyk et al. 2023). Formally, the GGI network is denoted as G=(V,E), where V stands for the set of nodes, and E represents the set of edges. Each node $v_{i}\in V$ is a protein-coding gene and each edge $(v_{i},v_{j})\in E$ stands for an interaction between genes *v*_*i*_ and *v*_*j*_ with a STRING score greater than 0.8. Note that we add a self-loop (*v*_*i*_, *v*_*i*_) to each node $v_{i}\in V$ in the network. Finally, we obtain a GGI network encompassing 19,836 genes and 250,236 interactions.

### PRS-Net

#### Graph neural network

We utilize a GNN to integrate gene-level PRSs leveraging GGIs (Fig. 1C). In this study, we specifically opt for a GIN (Xu et al. 2018) owing to its demonstrated theoretical and empirical expressiveness. In particular, given input PRS features ***h***_*i*_ ∈ ***H*** for $v_{i}\in V$, where $H\in R^{|V|\times 11}$ and |V| is the number of genes in G, we first perform feature embedding:

(1) $$H^{0}=\mathrm{ML}P^{0}(H),$$

where $H^{0}\in R^{|V|\times D}$ and *D* is the dimension of hidden features. Next, we apply multiple GIN layers to iteratively update the features of each node by aggregating neighboring information, as depicted below:

(2) $$h_{i}^{k}=\mathrm{ML}P^{k}((1+\epsilon^{k})\cdot h_{i}^{k-1}+\underset{v_{j}\in N(v_{i})}{\sum }h_{j}^{k-1}),$$

where $h_{i}^{k}$ is the hidden feature of *v*_*i*_ at the *k*th layer, $N(v_{i})$ stands for the neighbors of *v*_*i*_ in the GGI network, MLP^*k*^ is the MLP at the *k*th layer, and ϵ is a learnable variable. After *K* steps of messaging passing, each gene effectively encapsulates the PRS information within its *K*-hop neighborhood.

#### Attentive readout module

Following the GNN operation, we compute a global representation for each sample using an attentive readout module shown as follows:

(3) $$\begin{array}{rl} & h_{G}=\mathrm{Attentive}\;\mathrm{readout}(Q,K,V),\\ & h_{G}=A\cdot V,\\ & A=\mathrm{Sigmoid}(Q\cdot K),\\ & K=H^{k}\cdot W_{K},V=H^{k}\cdot W_{V}, \end{array}$$

where ***W***_*K*_ ∈ ℝ^*D*×*D*^ and ***W***_*V*_ ∈ ℝ^*D*×*D*^ are trainable key (i.e., ***K***) and value (i.e., ***V***) matrices, respectively; ***Q*** ∈ ℝ^1×*D*^ is the trainable query vector; $A\in R^{1\times |V|}$ are the attention scores; and $h_{G}\in R^{1\times D}$ is the global representation. Note that a higher attention score indicates a greater disease-relevance of the corresponding gene.

With the global representation $h_{G}$, we next employ an MLP to make the final prediction denoted as $P\hat{R}S$:

(4) $$P\hat{R}S=\mathrm{MLP}(h_{G}).$$

Additionally, we implement a mixture-of-expert module (Masoudnia and Ebrahimpour 2014) to handle cross-ancestry prediction. In particular, we introduce a separate attentive readout module for each ancestry (referred to as ancestry-specific attention module), and it is activated only if the input sample is with a matching ancestral origin. For instance, when dealing with individuals of EUR ancestry, we calculate the ancestry-specific global representation as follows:

(5) $$h_{G}^{\mathrm{EUR}}=\mathrm{Attentive}\;\mathrm{readout}(Q^{\mathrm{EUR}},K^{\mathrm{EUR}},V^{\mathrm{EUR}}).$$

The ancestry-specific readout module is designed to capture ancestry-specific disease associations. We also introduce a shared readout module to learn general genetic patterns independent with ancestries:

(6) $$h_{G}^{\mathrm{PH}}=\mathrm{Attentive}\;\mathrm{readout}(Q^{\mathrm{PH}},K^{\mathrm{PH}},V^{\mathrm{PH}}).$$

The final global representation is given by combining the aforementioned two representations:

(7) $$h_{G}=h_{G}^{\mathrm{EUR}}+h_{G}^{\mathrm{PH}}.$$

We refer to the single-ancestry model as PRS-Net and the multiancestry variation as PRS-Net_MA_.

### Model evaluation

We constructed target cohorts for eight diseases, including AD, AF, RA, MS, UC, asthma, MI, and CAD, and two quantitative traits, including height and BMI, based on the UKBB database (Sudlow et al. 2015). The ICD-10 codes (World Health Organization et al. 1992) were utilized to define disease phenotypes (Supplemental Table S1). Quantitative traits (height: Data Field 12144; BMI: Data Field 21002) were averaged for individuals across multiple instances.

In our experiments, we mainly focused on EUR individuals owing to the insufficient sample size of non-Europeans for certain diseases (Supplemental Table S2). After stringent quality controls (QCs) following the best practice (Supplemental Methods; Choi et al. 2020), each disease data set (target cohort) consisted of about 390,000 individuals. To prevent information leakage, we carefully chose GWAS not including UKBB samples in gene-level PRS calculation. GWAS summary statistics for diseases and traits are available from their original publications (Wood et al. 2014; Locke et al. 2015; The CARDIoGRAMplusC4D Consortium 2015; Christophersen et al. 2017; De Lange et al. 2017; International Multiple Sclerosis Genetics Consortium et al. 2019; Wightman et al. 2021; Ishigaki et al. 2022; Zhou et al. 2022).

For each target cohort, we randomly partitioned it into training, validation, and test sets with a ratio of 8:1:1. The validation data set was used for early stopping, and the final performance was evaluated on the test data set. More PRS-Net training details can be found in Supplemental Methods.

Following the best practice (Choi et al. 2020), we also implemented four traditional linear PRS models, including two C + T-based methods (PLINK [Purcell et al. 2007] and PRSice2 [Choi and O'Reilly 2019]), lassosum2 (Privé et al. 2022), and LDpred2 (LDpred2-auto, LDpred2-grid, and LDpred2-inf) (Privé et al. 2021), as well as two nonlinear models, including MLP and XGBoost, as our baseline methods. More implementation details can be found in Supplemental Methods. LD estimation used in baselines was the same as that adopted in PRS-Net.

We adopted two metrics, AUROC and AUPRC, to measure the performance for disease classification and explained variance and *R*^2^ for quantitative trait regression. Performance of all methods was estimated based on six independent runs with different random seeds for both data partitioning and model initialization.

### Software availability

The source code and the tutorial of PRS-Net are available at GitHub (https://github.com/lihan97/PRS-Net) and as Supplemental Code.

## Supplemental Material

Supplement 1

Supplement 2

Supplement 3

Supplement 4
