# Supplementary material for: Modeling gene interactions in polygenic prediction via geometric deep learning
Source: Genome Res. 2025 Jan;35(1):178–87. doi: 10.1101/gr.279694.124 (PMC11789630; doi:10.1101/gr.279694.124)
Supplement: Supplement 1 [file Supplemental_Material.pdf]

Supplemental Methods

Quality controls

GWAS data quality controls. In processing the GWAS summary statistics data, several steps were taken following the best practice (Choi et al., 2020). Variants with a minor allele frequency (MAF) less than 0.1% and an imputation information score below 0.3 were excluded. In cases where variants had allelic inconsistencies between the base and target data, strand-flipping was applied if the inconsistency could be resolved; otherwise, the non-resolvable variants were removed. Duplicate variants were also excluded to retain only one instance of each. Ambiguous variants were entirely removed from the dataset.

Target data quality controls. In processing the UKBB individual-level data, we implemented several filtering steps following the best practice (Choi et al., 2020). We excluded variants with a genotyping rate below 1%, a minor allele frequency lower than 0.1%, or those not conforming to Hardy-Weinberg equilibrium (*P*-value less than 1×10^{-10}). Duplicate variants were also removed to retain only one instance of each. Additionally, individuals with discrepancies between their reported sex and genetic sex were removed. Individuals that have a first or second degree relative (π $>$ 0.125) in the cohort were removed. For each disease phenotype, variants that exhibited a statistically significant difference in missing rate between cases and controls (with a *P*-value less than 1×10^{-5}) were identified using Fisher's exact test and subsequently removed from the analysis.

PRS-Net training details

PRS-Net was implemented in PyTorch (Paszke et al., 2017) version 1.13.1 and DGL (Wang et al., 2019) version 1.1.0 with CUDA version 11.6 and Python 3.7.16. We implemented a one-layer graph isomorphism network (Xu et al., 2018) (GIN) with a hidden size of 64. A multi-layer perceptron is employed as a predictor. The attentive readout module in PRS-Net differs from the attention layer commonly used in transformer architectures.

In PRS-Net, the attentive readout module assigns attention scores to individual nodes within the gene-gene interaction (GGI) network. Subsequently, a sum of the node embeddings weighted by their attentions is computed to derive the graph-level embeddings for PRS prediction. Therefore, our attentive readout module employs a single attention operation with a single head. While a multi-head attention mechanism similar to that is used in transformer could potentially enhance performance, we leave this as future work. In PRS-Net, the hidden dimension D is set to 64. Unlike the attention layer in transformer, which scales the attention weights by D, our attentive readout module does not require this scaling factor. The scaling of transformer's attention mechanism aims to stabilize the gradients and control the variance in dot product between the query and key matrices. This ensures that the magnitude of attention weights remains consistent across different values of D, facilitating effective learning and optimization in large-scale models with multiple attention layers. Given that our attentive readout module conducts only a single attention operation, the scaling by D here is not necessary.

We used a cross-entropy loss function for binary phenotypes and mean squared error as the objective function for quantitative traits. To address sample imbalance (i.e., less disease cases than healthy controls) and ensure effective model training, we implemented a balanced sampling strategy during the training process. Specifically, in each training step, we randomly sampled an equal number of cases and controls to construct each training batch. To allow the dataloader to keep sampling batches until the model converges, we set the number of samples to a large

value. For example, by setting n_samples to 10,000,000, the dataloader can generate a total of 10,000,000/batch_size batches during training. The AdamW optimizer with a learning rate of 1×10^{-4} was adopted to train the model. Training was performed with a batch size of 512 over a total of 20,000 steps, utilizing a single Nvidia A100 GPU.

Implementation of baseline methods

For PLINK, PRSice, LDPred-2, and lassosum2, we followed the implementation detailed in the PRS tutorial (https://choishingwan.github.io/PRS-Tutorial/). We used BOLT-LMM-inf to generate the best linear unbiased prediction (BLUP) estimates following BOLT-LMM manual (https://alkesgroup.broadinstitute.org/BOLT-LMM/BOLT-LMM_manual.html). For the Multi-Layer Perceptron (MLP), we implemented a three-layer architecture with a hidden size of 64, utilizing ReLU activation and batch normalization. The learning rate was set to 0.001. For XGBoost, we configured the number of gradient-boosted trees to 500 and the learning rate to 0.01, keeping all other hyper-parameters at their default settings. Both MLP and XGBoost took GWAS variants as inputs. We selected the best GWAS P-values (0.001, 0.0001, and 0.00001) based on performance on the validation set and reported the results on the test set.

Supplemental Figures

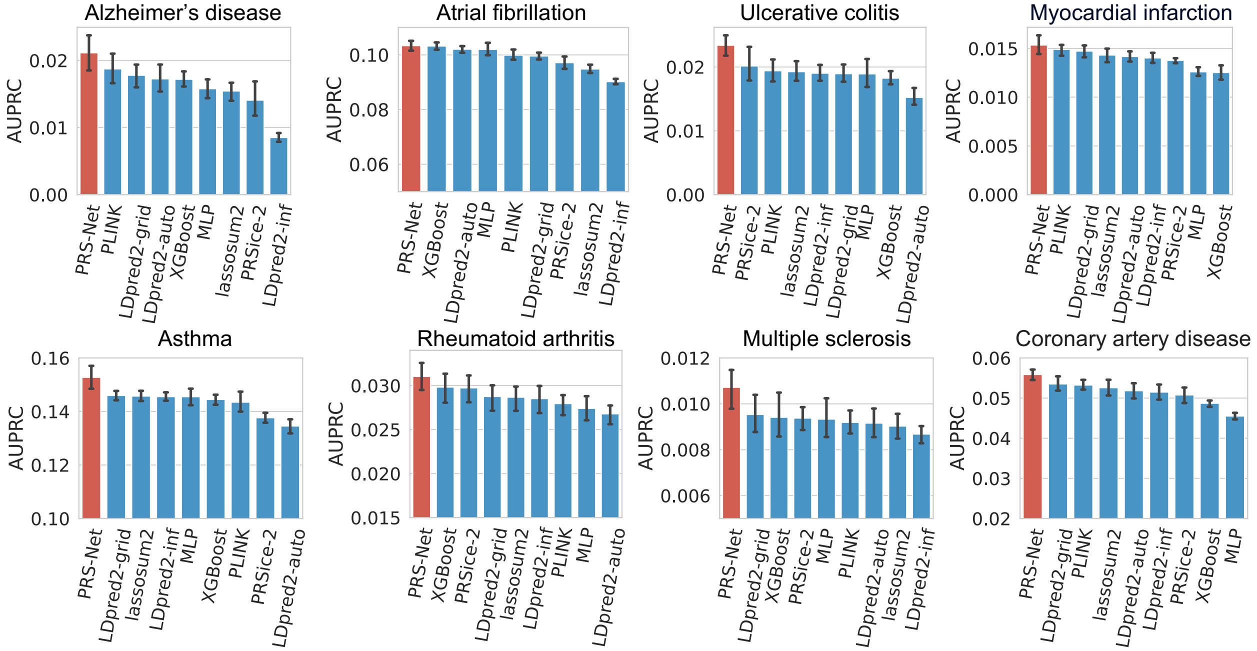

Supplemental Fig. S1: Prediction performance evaluation based on the area under the precision-recall curve (AUPRC) for different diseases $(41, 175$ test samples in total). The bar plot and error bar denote the mean and standard error, respectively. The training, validation, and testing procedure was conducted for six repeats with different random seeds for each model and each disease.

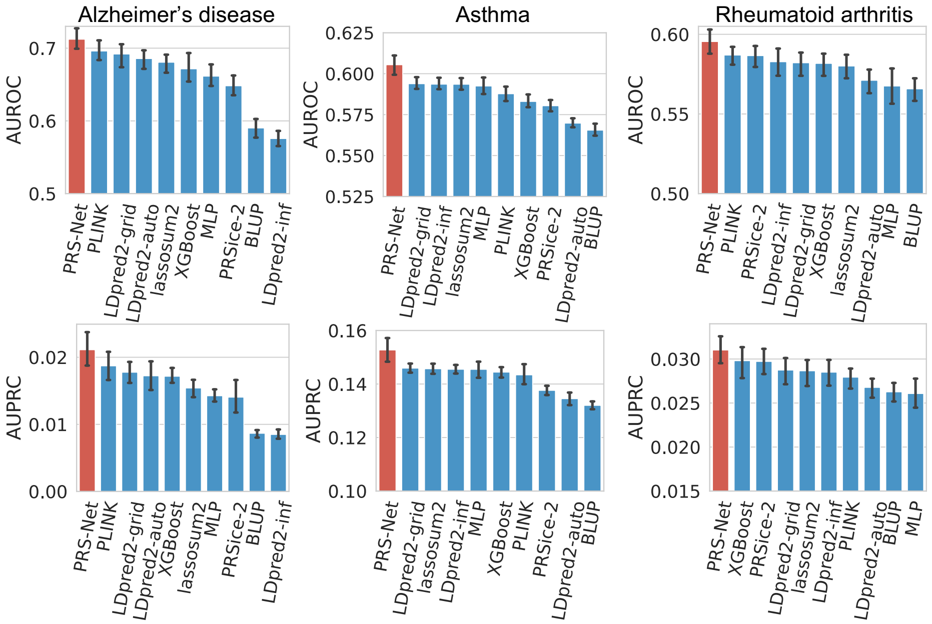

Supplemental Fig. S2: Additional prediction evaluation for diseases based on the area under the receiver operating characteristic curve (AUROC) and the area under the precision-recall curve (AUPRC) ($41,175$ test samples in total). The bar plot and error bar indicate the mean and standard error, respectively. The training, validation, and testing procedure was conducted for six repeats with different random seeds for each model and each disease.

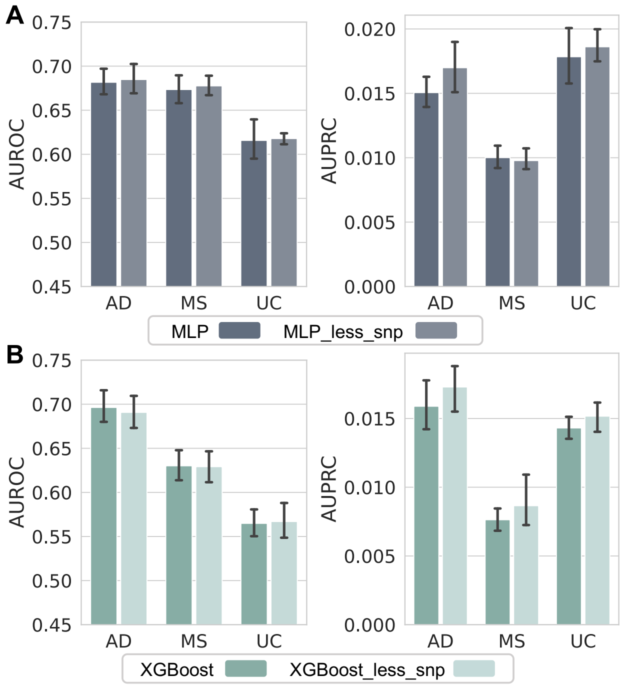

Supplemental Fig. S3: Prediction comparison for MLP_less_snp and XGBoost_less_snp. The bar plot and error bar denote the mean and standard error, respectively. The training, validation, and testing procedure was carried out for six repeats with different random seeds for each model and each disease. AD, Alzheimer's disease; MS, multiple sclerosis; UC, ulcerative colitis; AUROC, the area under the receiver operating characteristic curve; AUPRC, the area under the precision-recall curve.

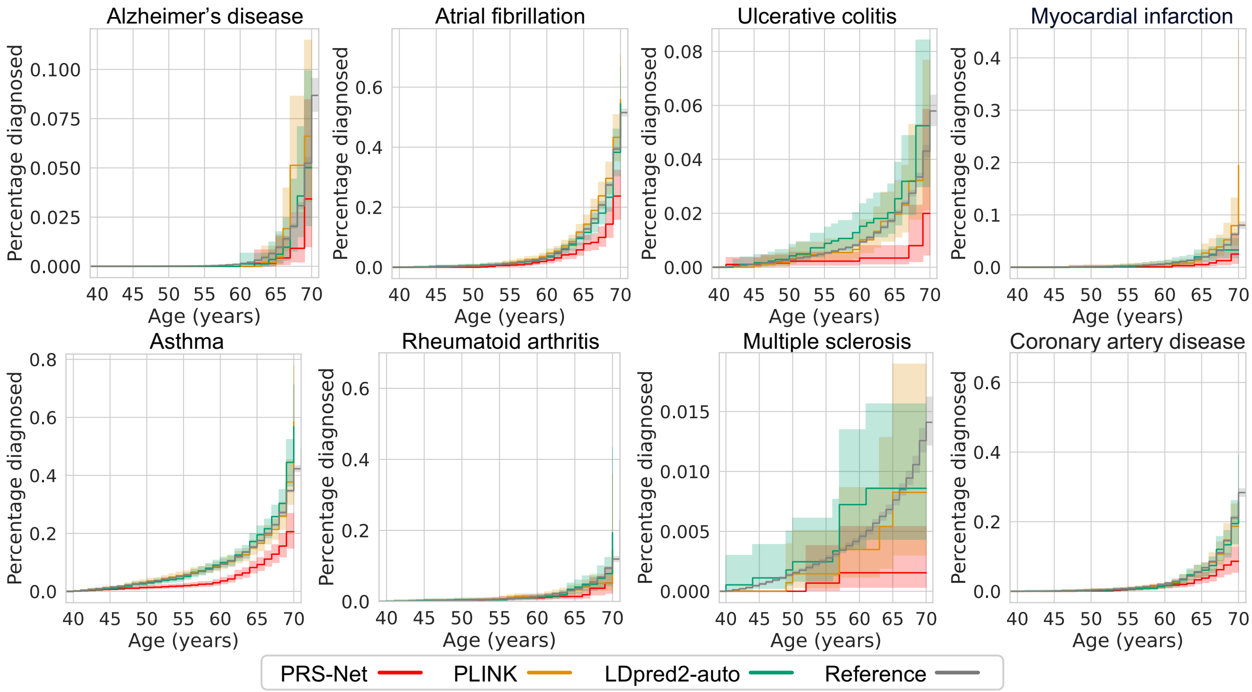

Supplemental Fig. S4: The cumulative incidence plots of low-risk individuals (with the lowest 5% PRSs) determined by PRS-Net and baseline methods. Each plot illustrates the estimated percentage of individuals diagnosed with a specific disease at different ages. We provide cumulative incidence plots for the original datasets as references. The line plot and shaded area represent the mean and standard error, respectively.

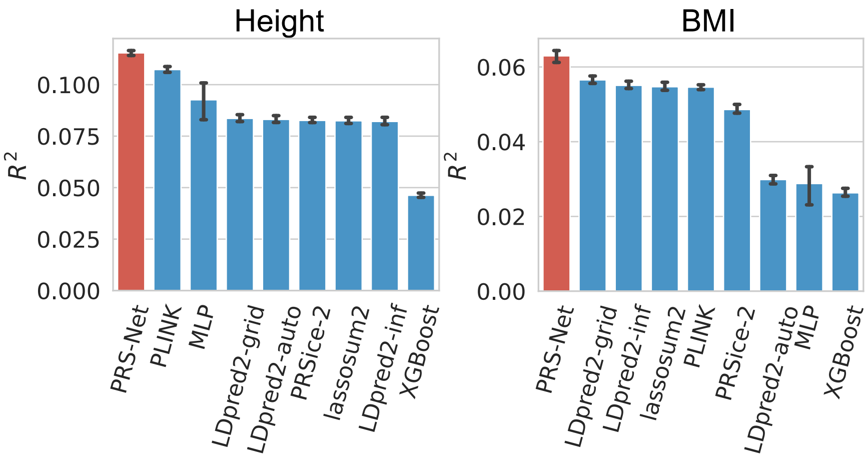

Supplemental Fig. S5: Prediction performance evaluation for quantitative traits ($41,028$ and $40,411$ test samples for height and BMI, respectively). Performance was measured in R^2. The bar plot and error bar denote the mean and standard error, respectively. The training, validation, and testing procedure was conducted for six repeats with different random seeds for each model and each trait. BMI, body mass index.

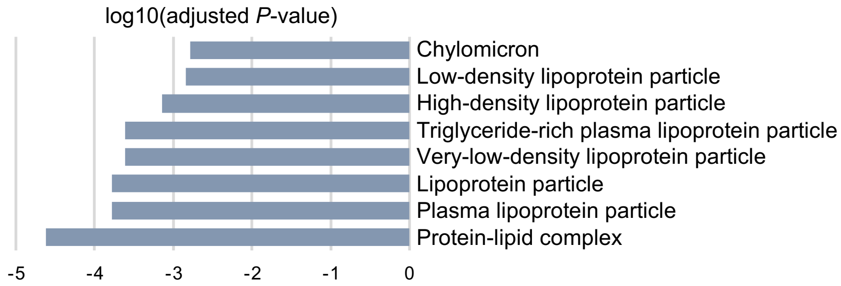

Supplemental Fig. S6: Gene set enrichment analysis (GSEA) for Alzheimer's disease genes identified by PRS-Net, using gene ontology (GO) dataset as a reference. GO terms with adjusted $P < 0.05$ (Benjamini-Hochberg correction) were shown.

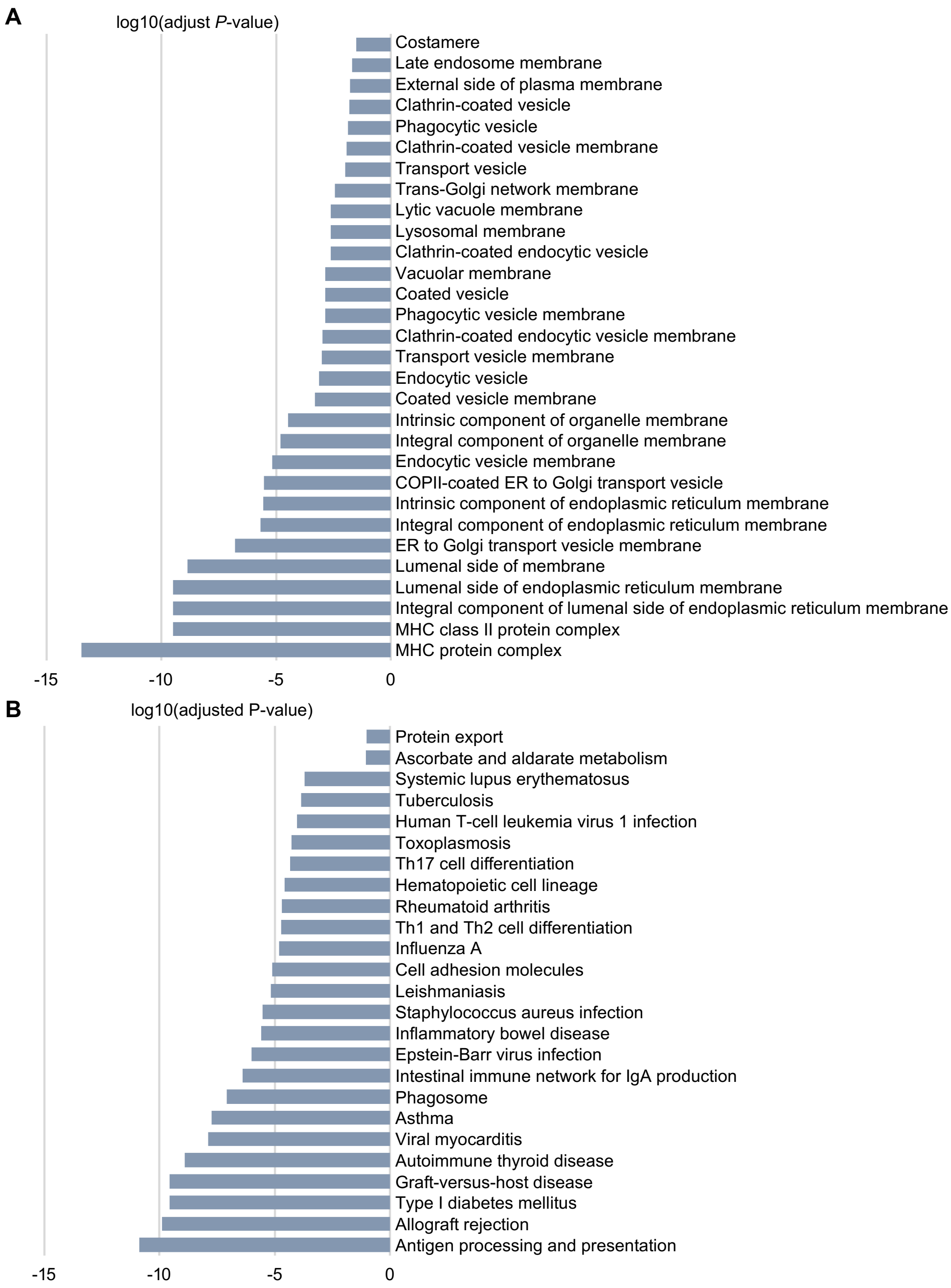

Supplemental Fig. S7: Gene set enrichment analysis (GSEA) for multiple sclerosis genes identified by PRS-Net, using (A) gene ontology (GO) and (B) KEGG datasets as references. Terms with adjusted $P < 0.05$ (Benjamini-Hochberg correction) were shown.

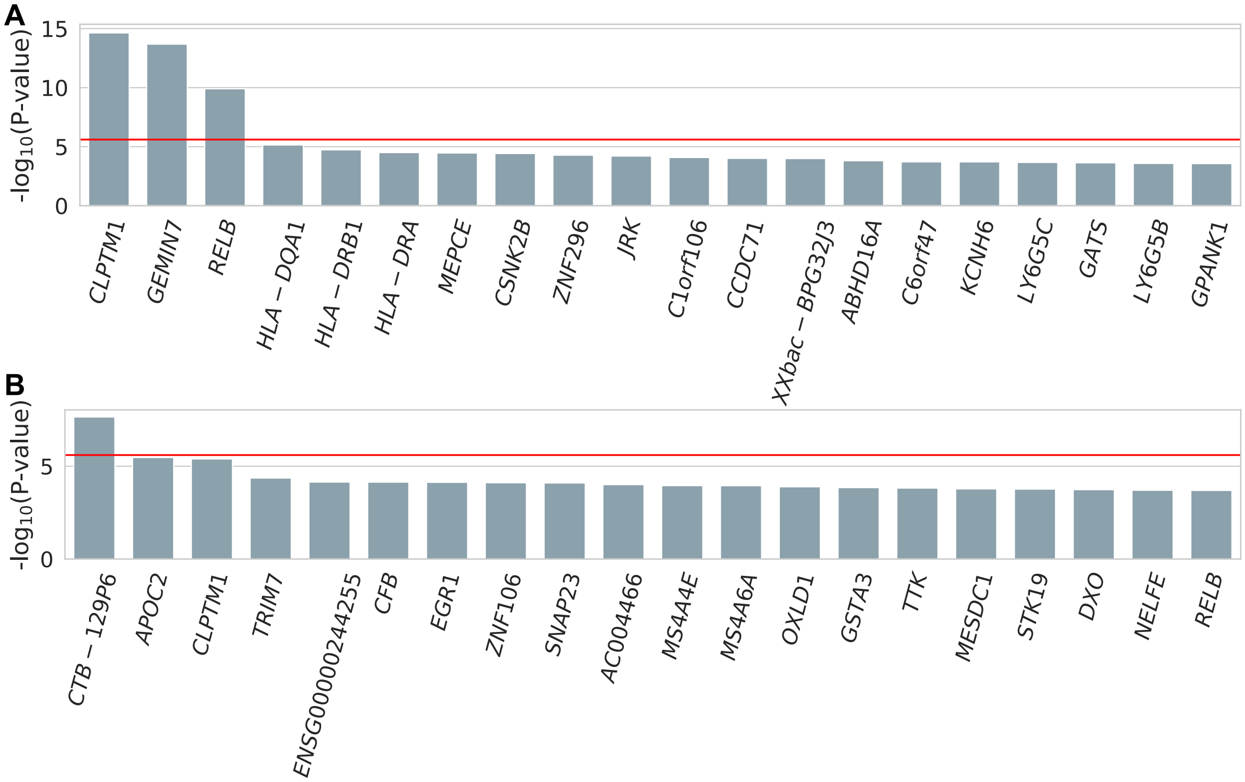

Supplemental Fig. S8: Disease genes identified by a variation of PRS-Net without gene-gene interaction network (PRS-Net-noPPI). (A-B), Top 20 genes ranked by P-value based on the Mann-Whitney U test for Alzheimer's disease (A) and multiple sclerosis (B), respectively. The red line denotes the Bonferroni significance level.

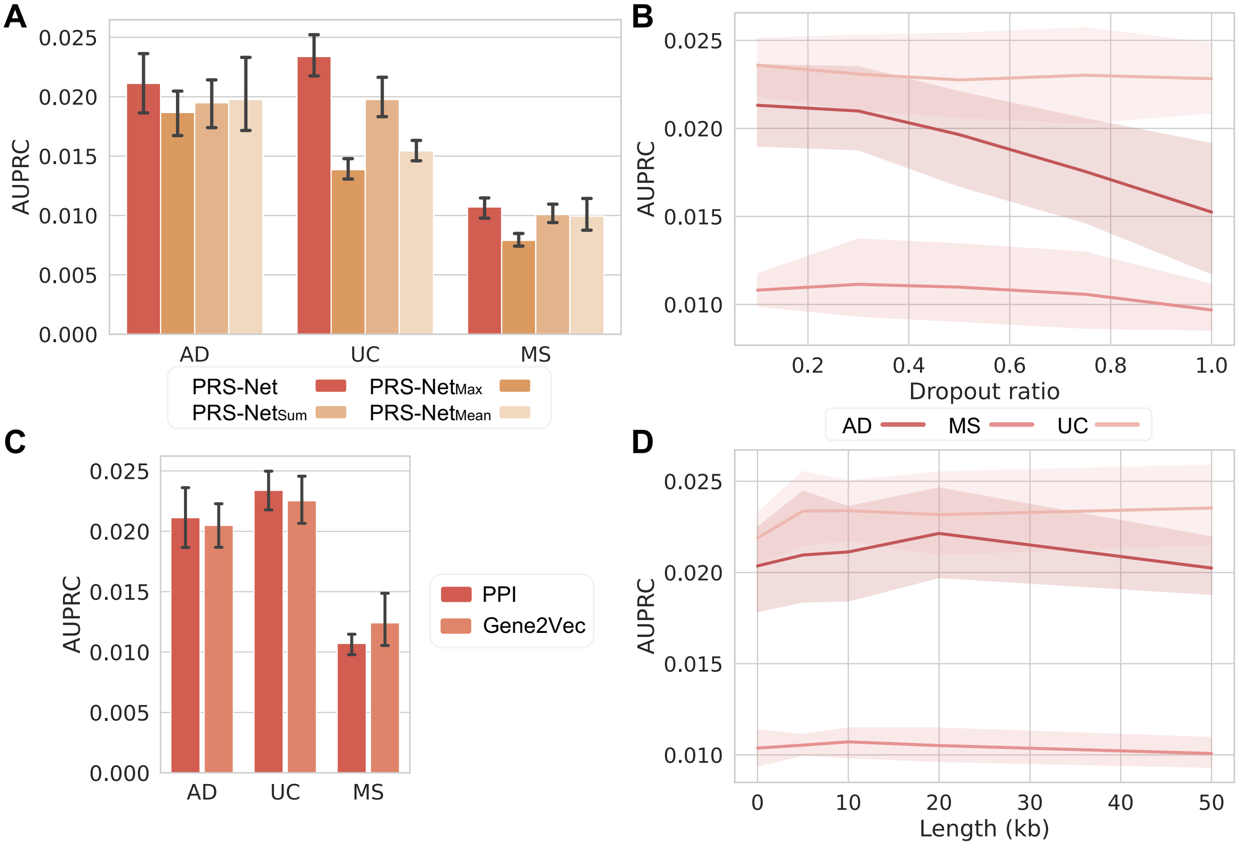

Supplemental Fig. S9: The ablation results for PRS-Net in AUPRC. (A) The performance comparison between PRS-Net and its variations. The bar plot and error bar denote the mean and standard error, respectively. (B) The performance of PRS-Net with PPI dropout. The line plot and shaded area denote the mean and standard error, respectively. (C) Comparison results of PRS-Net with different GGI networks. (D) The prediction performance of PRS-Net with different extension lengths. AD, Alzheimer's disease; MS, multiple sclerosis; UC, ulcerative colitis; AUPRC, the area under the precision-recall curve; PPI, protein-protein interaction.

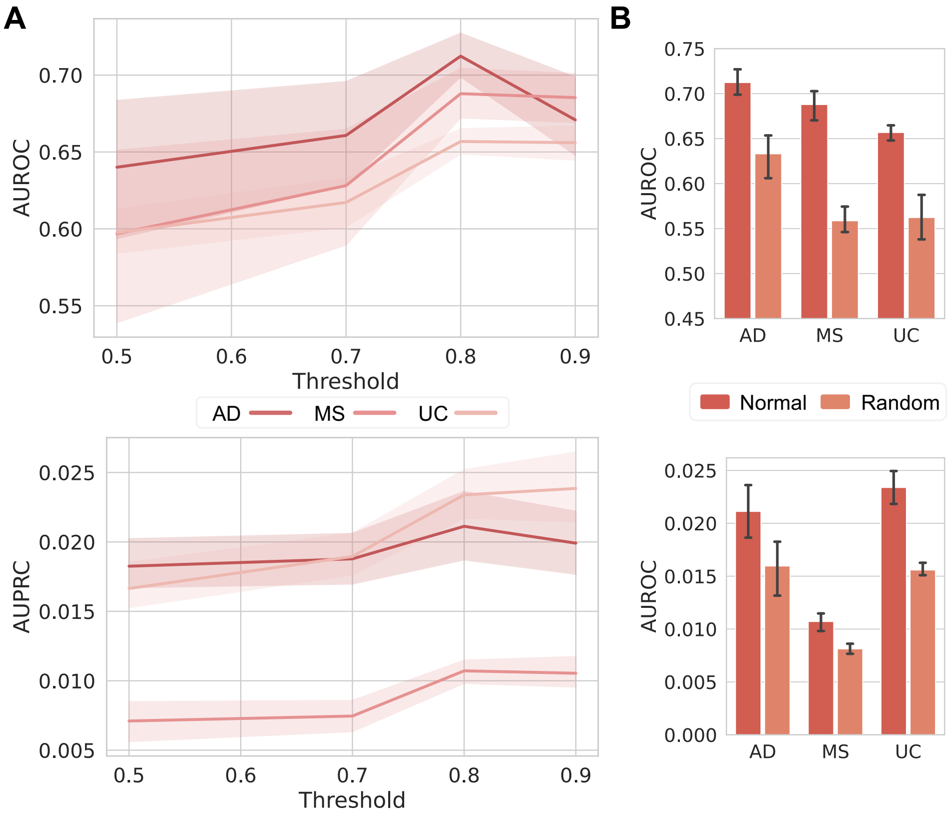

Supplemental Fig. S10: The ablation results on the PPI network. (A) The performance of PRS-Net with different thresholds for PPI filtering. The line plot and shaded area denote the mean and standard error, respectively. (B) The performance comparison of PRS-Net with original and random PPI networks. The bar plot and error bar denote the mean and standard error, respectively. AD, Alzheimer's disease; MS, multiple sclerosis; UC, ulcerative colitis; AUROC, the area under the receiver operating characteristic curve; AUPRC, the area under the precision-recall curve.

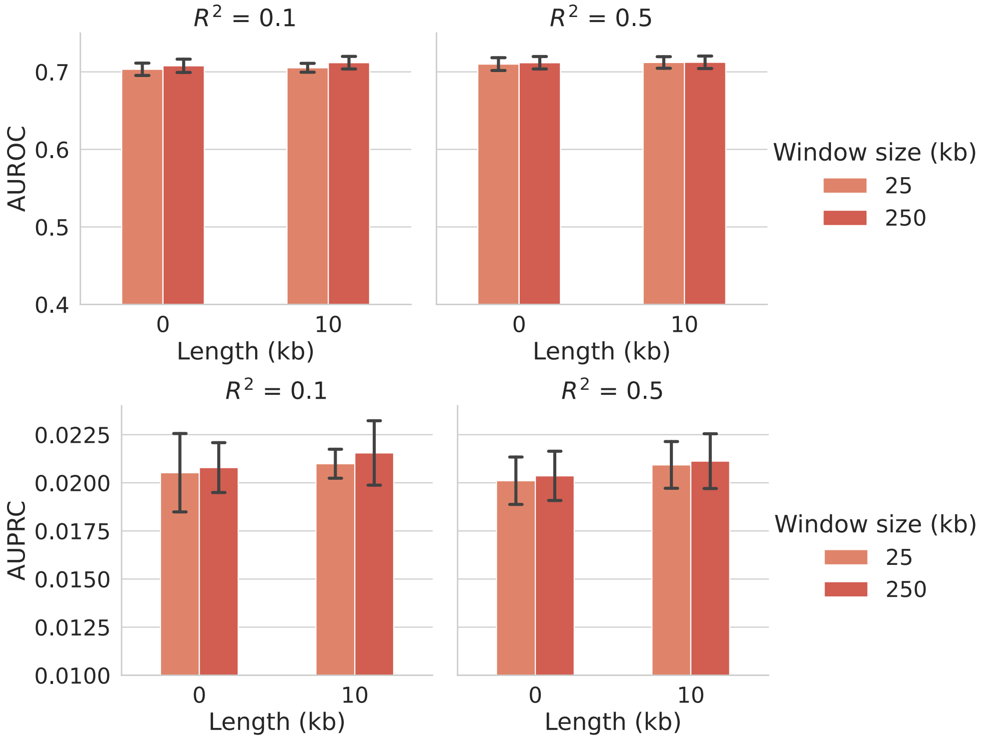

Supplemental Fig. S11: The ablation results on different model hyperparameters. The performance comparison of PRS-Net with different values of R^2, L, and window size on Alzheimer's disease. The bar plot and error bar denote the mean and standard error, respectively. The training, validation and testing procedure was conducted for six repeats with different random seeds. AUROC, the area under the receiver operating characteristic curve; AUPRC, the area under the precision-recall curve.

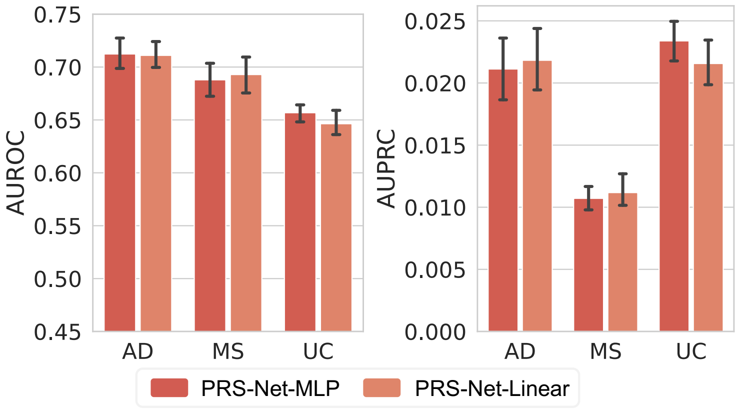

Supplemental Fig. S12: The ablation results on the predictor. The performance comparison of PRS-Net with a linear predictor (PRS-Net-Linear) and with a multiple-layer perceptron (MLP) predictor (PRS-Net-MLP). The bar plot and error bar denote the mean and standard error, respectively. The training, validation and testing procedure was conducted for six repeats with different random seeds. AD, Alzheimer's disease; MS, multiple sclerosis; UC, ulcerative colitis; AUROC, the area under the receiver operating characteristic curve; AUPRC, the area under the precision-recall curve.

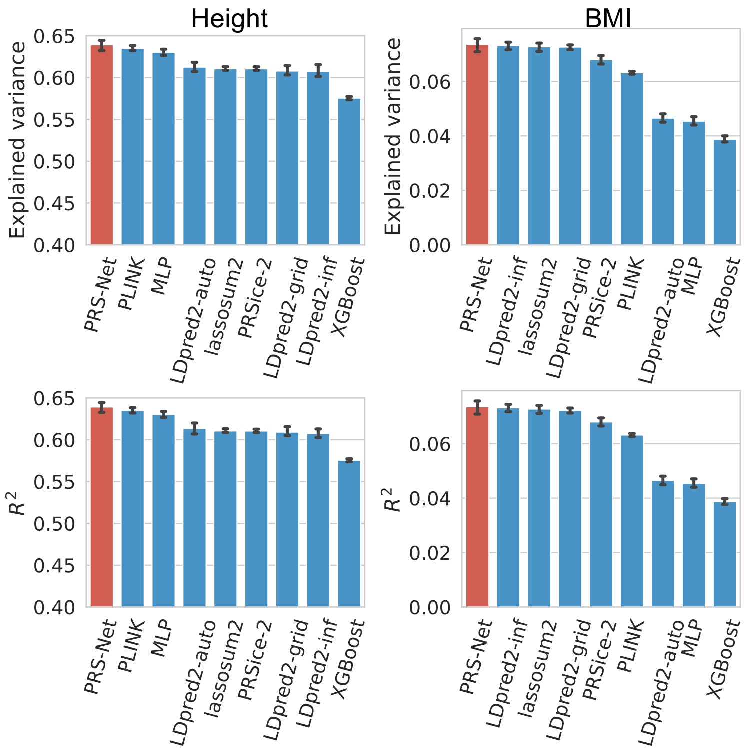

Supplemental Fig. S13: Prediction performance evaluation for quantitative traits including age, sex, and principal components (PCs) as covariates ($41,028$ and $40,411$ test samples for height and BMI, respectively). Performance was measured in explained variance and R^2. The bar plot and error bar denote the mean and standard error, respectively. The training, validation, and testing procedure was conducted for six repeats with different random seeds for each model and each trait. BMI, body mass index.

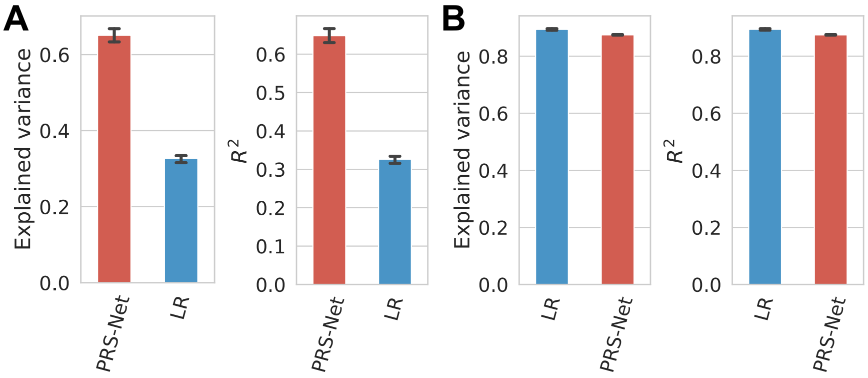

Supplemental Fig. S14: Prediction performance evaluation for simulation dataset ($41, 028$ test samples in total). Performance was measured in explained variance and R^2. (A) Prediction performance on the non-linear simulation dataset. (B) Prediction performance on the linear simulation dataset. The bar plot and error bar denote the mean and standard error, respectively. The training, validation, and testing procedure was conducted for six repeats with different random seeds for each model. LR, linear regression.

Supplemental Tables

Supplemental Table S1: ICD-10 codes used to define different diseases.

**Phenotype**	**ICD-10**
Alzheimer’s disease	F00/G30
Atrial fibrillation	I48
Ulcerative colitis	K51/M07.5/M09.2
Asthma	J45/J46
Rheumatoid arthritis	M05/M06/M08.0
Multiple sclerosis	G35
Myocardial infarction	I21.9
Coronary artery disease	I25.1

Supplemental Table S2: Summary statistics of disease cohorts across multiple ancestry groups, including Western European (EUR), South Asian (SAS), and African (AFR) ancestry. Abbreviations: N (number), POS (positive), and NEG (negative).

Phenotype	N.POS.EUR (%)	N.NEG.EUR	N.POS.SAS (%)	N.NEG.SAS	N.POS.AFR (%)	N.NEG.AFR
Alzheimer's disease	2310 (0.59%)	388549	41 (0.47%)	8772	53 (0.60%)	8796
Atrial fibrillation	28754 (7.36%)	362105	374 (4.24%)	8439	266 (3.00%)	8583
Ulcerative colitis	4293 (1.10%)	386566	139 (1.58%)	8674	51 (0.58%)	8798
Multiple sclerosis	1742 (0.45%)	389117	8 (0.09%)	8805	16 (0.18%)	8833
Asthma	41443 (10.60%)	349416	1239 (14.06%)	7574	1048 (11.84%)	7801
Rheumatoid arthritis	7566 (1.94%)	383293	242 (2.75%)	8571	186 (2.10%)	8663
Myocardial Infarction	4205 (1.08%)	349416	167 (1.89%)	7574	69 (0.78%)	7801
Coronary artery disease	14582 (3.73%)	383293	603 (6.84%)	8571	181 (2.05%)	8663

Supplemental Table S3: Alzheimer's disease genes identified by PRS-Net.

Supplemental Table S4: Multiple sclerosis genes identified by PRS-Net.

References

Shing Wan Choi, Timothy Shin-Heng Mak, and Paul F O'Reilly. Tutorial: a guide to performing polygenic risk score analyses. Nature protocols, 15(9):2759–2772, 2020.

Adam Paszke et al. Automatic differentiation in pytorch. In NIPS-W, 2017.

Minjie Wang et al. Deep graph library: A graph-centric, highly-performant package for graph neural networks. arXiv preprint arXiv:1909.01315, 2019.

Keyulu Xu, Weihua Hu, Jure Leskovec, and Stefanie Jegelka. How powerful are graph neural networks? In International Conference on Learning Representations, 2018.
